# Clinical deterioration after implantation of an interatrial shunt device: case report of an unexpected aetiology

**DOI:** 10.1093/ehjcr/ytab009

**Published:** 2021-02-08

**Authors:** Richard J Nies, Henning Guthoff, Navid Mader, Roman Pfister

**Affiliations:** 1 Department of Cardiology, Heart Center, University of Cologne, Kerpener Str. 62, D-50937, Cologne, Germany; 2 Department of Cardiothoracic Surgery, Heart Center, University of Cologne, Kerpener Str. 62, D-50937, Cologne, Germany

A 76-year-old patient with heart failure with preserved ejection fraction underwent interatrial shunt device (Corvia Medical IASD^®^) implantation to decrease left heart filling pressures. Five weeks later the patient was readmitted with worsening dyspnoea and ankle oedema. Transthoracic echocardiography showed preserved left- and right ventricular ejection fraction without relevant valvular heart disease. However, a new right atrial mass adjacent to the IASD was detected and confirmed by transoesophageal echocardiography (*Panels A* and *B*; *Videos 1–3*) and computed tomography (*Panel C*). Device-associated thrombus extending along the shunt flow into the inferior vena cava was suspected. Intravenous anticoagulation and subsequent systemic fibrinolysis failed to cause thrombus regression. After further clinical worsening a heart-team decision for surgical thrombectomy and IASD removal was made (*Panel D*). Post-operative high-dose catecholamine therapy caused severe mesenterial ischaemia with septic shock and fatal multiorgan failure.

**Figure:**
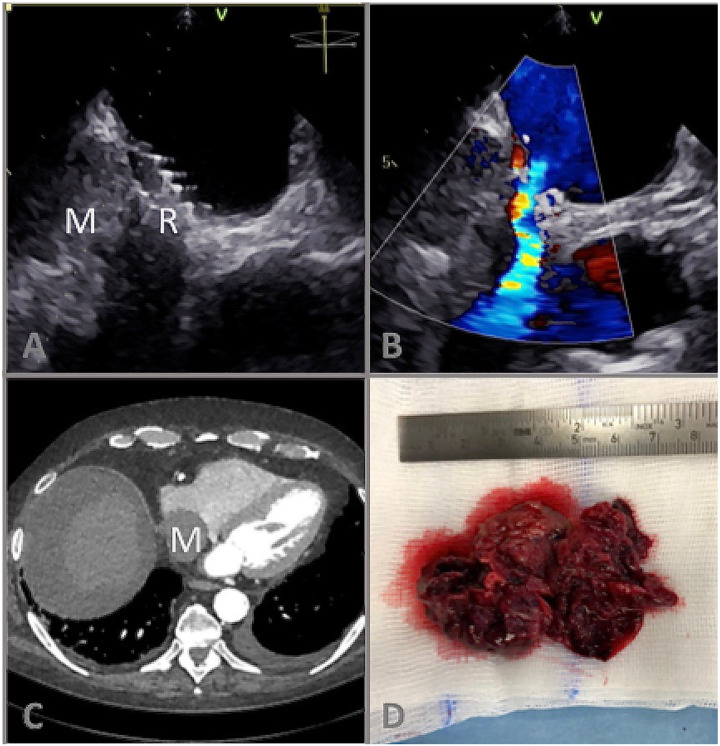


This case illustrates differential diagnosis of worsening heart failure in patients following IASD implantation. Decompensation of pre-existing left-sided heart failure and, alternatively, right heart failure resulting from atrial left-to-right shunt should be considered. Finally, mechanical obstruction due to a device-associated thrombus might be causative. So far, one case of suspected thrombus formation during IASD implantation has been reported, but device-associated thrombus is reported in up to 1% after atrial septal occluder implantation. In our case, thrombus originated from infiltrative hepatocellular carcinoma, which was diagnosed by histopathology, computed tomography scans and markedly elevated alpha-fetoprotein. Interatrial shunt device was not causally involved. Rare severe secondary Budd-Chiari syndrome caused by tumorous compression or infiltration of the hepatic outflow has been reported.

(*Panel A*) B-Mode transoesophageal echocardiography of a right atrial mass (M) in close relation to the atrial flow regulator (interatrial shunt device) (R); (*Panel B*) Colour Doppler showing left-to-right shunt flow via interatrial shunt device; (*C*) Computed tomography scan showing the right atrial mass (M); (*D*) surgical preparation specimen.

## Supplementary material

Supplementary material is available at *European Heart Journal - Case Reports* online.

**Slide sets:** A fully edited slide set detailing these cases and suitable for local presentation is available online as Supplementary data.

**Consent:** The authors confirm that written consent for submission and publication of this case report including images and associated text has been obtained from the patient's next of kin in line with COPE guidance.

